# Decreased Levofloxacin Susceptibility in *Haemophilus influenzae* in Children, Hong Kong

**DOI:** 10.3201/eid1011.040055

**Published:** 2004-11

**Authors:** Pak-Leung Ho, Kin Hung Chow, Gannon C. Mak, Kenneth W. Tsang, Yu Lung Lau, Alex Y. Ho, Eileen L. Lai, Susan S. Chiu

**Affiliations:** * University of Hong Kong, Hong Kong Special Administrative Region, China

**Keywords:** Haemophilus influenza, drug resistance, microbial, anti‑infective agents, fluoroquinolone, pulsed-field gel electrophoresis, dispatch

## Abstract

Among 563 strains of *Haemophilus influenzae* from young children in Hong Kong, 5 (0.9%) had decreased susceptibility to quinolones. The five strains had a Ser-84-Lys or Asp-88-Asn substitution in GyrA. Pulsed-field gel electrophoresis showed that the isolates are genetically diverse.

Although fluoroquinolone-resistant *Haemophilus influenzae* were reported a decade ago (*1*), this resistance phenotype has remained rare (*2*). In general, resistance to fluoroquinolone is chromosome-mediated, involves mutations in one or both target genes encoding DNA gyrase and topoisomerase IV, and tends to develop in a stepwise manner. The MICs of various antimicrobial agents for such mutant strains increases with each additional mutation in the target sites. In *H. influenzae*, high-level resistance to the fluoroquinolones generally occurs in the presence of mutations involving both *gyrA* and *parC*. In *H. influenzae*, *gyrA* is the primary target for fluoroquinolones because *gyrA* mutations have generally arisen before *parC* mutations in resistant clinical isolates (*2*). First-step *gyrA* mutants showed reduced susceptibility to fluoroquinolones, but the MICs remain in the susceptible range. Resistance mechanisms in these isolates will be undetected if the fluoroquinolones' susceptibility was determined and the results interpreted according to the current breakpoints. We assessed the susceptibility of *H. influenzae* nasopharyngeal isolates, obtained from children throughout Hong Kong, to nalidixic acid and levofloxacin.

## The Study

During the study period (December 1999 to June 2000), a total of 1,978 children, 2 to 6 years of age, were recruited from 79 daycare centers or kindergartens throughout Hong Kong. Details of the study population and the findings in relation to *Streptococcus pneumoniae* have been described (*3*). In brief, nasopharyngeal swab specimens were obtained from a predetermined number of children in each day care center or kindergarten. On average, 25 children (standard deviation [SD] 11) from each institute were examined. For isolation of *H. influenzae*, a previously described selective medium (chocolate gonococcal [GC] agar base with sheep blood, supplemented with 1% yeast autolysate and vancomycin 5 µg/mL, bacitracin 300 µg/mL, and clindamycin 1 µg/mL) was used for swab inoculation (*4*). Plates to which samples were added were incubated in 5% CO_2_ for <48 h. All isolates were identified by colony morphologic features, Gram stain, and requirement for both X and V factors.

The MICs of nalidixic acid, levofloxacin, ampicillin, and azithromycin were determined by the MIC microbroth dilution method with an in-house *Haemophilus* test medium broth (*5*) and interpreted according to National Committee for Clinical Laboratory Standards (NCCLS) guidelines (*6*). Quality control strains (*H. influenzae* ATCC 49247 and ATCC 49766) were included with each run. All isolates were tested for the production of β-lactamase by nitrocephin paper disks (Cefinase, BBL, Becton Dickinson Microbiology Systems, Franklin Lakes, NJ).

The subset of isolates with reduced susceptibility to levofloxacin was examined further by pulsed-field gel electrophoresis (PFGE) using *Sma*I for DNA digestion (*7*), and the results were interpreted according to Tenover et al. (*8*). The isolates were also examined for *gyrA* and *parC* mutations by using primers and methods described (*7*).

The median age (interquartile range) for these 1,978 children was 5.3 years (4.3-5.3 years); the mean age was 5 years. Approximately half of the children were boys (52.7%). Sixty-three percent of surveyed children had siblings <12 years of age; 277 (14%) of the children had an overcrowded living environment (living space <5.5 m^2^/person, according to the guideline of the Hong Kong Housing Authority). At the time of the survey, 103 (5.2%) of the 1,978 children were reported to be taking antimicrobial agents. In the 3 months before the study, 1,535 (77.6%) had visited their family doctor, and 63 (3.2%) had been hospitalized.

Overall, the carriage rate of *H. influenzae* was 28.5% (range 17%–42.1%). The MICs of nalidixic acid and levofloxacin for all isolates are shown in Table 1. Five (0.9%) isolates were resistant to nalidixic acid with MICs of 64 µg/mL to 128 µg/mL. The levofloxacin MICs of the same five isolates were 0.125 µg/mL, which is higher than the MICs (range 0.0019–0.06 µg/mL; mode 0.015) of the same antimicrobial drug for the nalidixic acid–sensitive isolates. Of the 563 isolates, 158 (28.1%) were β-lactamase–positive strains and thus were resistant to ampicillin. All isolates were susceptible to azithromycin with an MIC_50_ of 1 µg/mL and an MIC_90_ of 2 µg/mL.

**Table 1:** Levofloxacin and nalidixic acid MIC distributions of all *Haemophilus influenzae* strains

MIC (µg/mL)	No. of strains	
	Levofloxacin	Nalidixic acid
0.001875	1	0
0.00375	1	0
0.0075	24	0
0.015	464	0
0.03	66	0
0.06	2	0
0.125	5	0
0.5	0	105
1	0	303
2	0	147
4	0	2
8	0	1
64	0	4
>128	0	1

None of the five children with nalidixic acid–resistant *H. influenzae* had been previously hospitalized. All five children had been treated with antimicrobial drugs in the previous 3 months, and two were taking antimicrobial drugs at the time samples were obtained. The specific antimicrobial agents were unknown. Two children had asthma, and the remaining three children had no underlying diseases.

The quinolone resistance–determining regions of *gyrA* and *parC* for the eight isolates with resistance or reduced susceptibility to nalidixic acid (MIC > 4 µg/mL) or levofloxacin (MIC >0.06 µg/mL) were sequenced (Table 2). A Ser-84-Lys or Asp-88-Asn substitution was found in GyrA in all five isolates with resistance to nalidixic acid. No substitutions occurred in ParC. No amino acid substitution was found in either GyrA or ParC in the three isolates with reduced susceptibility to nalidixic acid (MIC 4–8 µg/mL). In pulsed-field gel electrophoresis analysis, the five nalidixic acid–resistant strains had distinct patterns and were unrelated.

**Table 2:** MIC and QRDR amino acid substitutions of *Haemophilus influenzae* ATCC 49247 and eight clinical isolates from young children^a^

Isolate	School	Sex/age (y)	MIC				Predicated QRDR amino acid changes	
			NA	LVX	AMP	AZI	GyrA	ParC
ATCC 49247	–	–	1	0.015	4	1	^80^PHGDSAVYDTIVR^92^	^80^PHGDSACYEAMVL^92^
G813	G8	M/6	4	0.03	1	2	None	None
G92	G9	M/4	4	0.06	0.25	1	None	None
D19	D1	M/6	8	0.06	0.25	1	None	None
L38	L3	F/5	64	0.125	0.5	1	D88N (gat to aat)	None
J517	J5	M/6	64	0.125	0.5	2	S84L (tcc to ttg)	None
M65	M6	F/6	64	0.125	128	1	S84L (ttc to tta)	None
B211	B2	F/4	64	0.125	0.25	1	S84L (tcc to tta)	None
R33	R3	F/5	128	0.125	0.25	1	S84L (ttc to tta)	None

^a^QRDR, quinolone resistance-determining region; NA, nalidixic acid; LVX, levofloxacin; AMP, ampicillin; AZI, azithromycin.

## Conclusions

Our data have shown, for the first time, resistance to quinolones among *H. influenzae* isolates in children. The finding is of clinical and public health concern, particularly in regions like Hong Kong where levels of antimicrobial resistance among respiratory pathogens are already high, and fluoroquinolone resistance in *S. pneumoniae* is emerging (*9**,**10*). The finding is also unexpected because fluoroquinolones are not approved for use among children in Hong Kong; such agents are not approved for children in the rest of the world as well. We believe that three potential explanations may account for the detection of fluoroquinolone resistance among children. First, nalidixic acid is approved to treat pediatric infections and is widely used in Hong Kong for outpatient and inpatient treatment of urinary tract infections and, occasionally, to treat shigellosis in children. In the Queen Mary Hospital, for instance, 13.5 and 10.0 defined daily doses per 100 pediatric admissions of nalidixic acid were used in 1999 and 2000, respectively (1 defined daily dose of nalidixic acid equals 4 g). In children, carriage of *H. influenzae* is common. The identified first-step mutant might be selected de novo when isolates colonizing the nasopharynx are exposed to the selection pressure from nalidixic acid. Second, use of fluoroquinolones in food animals is common in many Asian countries (*2*). Children could be exposed to residues of fluoroquinolones by consuming meat or dairy products from food animal previously fed antimicrobial agents from this group. At present, we do not know how exposure to residues of antimicrobial agents in food contributes to resistance (*11*). Presumably, the level of exposure from dietary source would be low. In the nasopharynx, the level of quinolone is approximately half the level it would be in the blood (*12*). If food levels of quinolones are controlled to within the acceptable minimum residual levels, the contribution from this route of potential exposure should be minimal. Finally, transmission from adults to children might have occurred in household settings. Although adult-to-child transmission appears to be uncommon, transmission of *H. influenzae* from child to adult or among siblings in household setting is well known (*13*). We do not have any comparative figures for isolates from adults. If adults are a source of the quinolone-resistant isolates, one would expect greater resistance rates in adults than children.

Detection of *H. influenzae* isolates from children with a first-step mutation in *gyrA* affects whether the fluoroquinolones should be approved for pediatric indications (*14*). So far, the main concerns among the scientific community have centered on the selection of fluoroquinolone-resistant pneumococci. Unlike adults, children frequently carry pneumococci in the nasopharynx and at high density. If the fluoroquinolones are used widely in children to treat infections such as salmonellosis, recurrent otitis media, and urinary tract infections, the selection of mutational resistance to the fluoroquinolones will likely occur more rapidly among children than among adults. Once resistance is selected, fluoroquinolone-resistant strains could disseminate rapidly and widely in the community by cross-transmissions in groups attending daycare centers and schools. Our finding thus highlights the need to monitor resistance not only among the infecting organisms being treated, but also the need to monitor colonizing bacteria in the same or other body sites that were also exposed to antimicrobial agents.

Our data have shown a low incidence (0.9%) of decreased levofloxacin susceptibility due to *gyrA* mutations among strains of *H. influenzae* isolated from children in Hong Kong. This finding warrants public health concern. Given that the fluoroquinolones might be increasingly used as a rescue therapy for certain pediatric infections that do not respond to other agents, surveillance of this type of resistance mechanism must be enhanced. In this regard, we have found that resistance to nalidixic acid (MIC > 64 µg/mL) or reduced susceptibility to levofloxacin (MIC > 0.125 µg/mL) might be useful surrogates. After we submitted this manuscript, similar observations on the laboratory detection of deceased susceptibility due to *gyrA* and *parC* mutations were reported (*15*); thus, our findings were corroborated.
